# Multi-trajectories of body mass index, waist circumference, gut microbiota, and incident dyslipidemia: a 27-year prospective study

**DOI:** 10.21203/rs.3.rs-4251069/v1

**Published:** 2024-04-19

**Authors:** Xiaofan Zhang, Fangxu Guan, Wanglong Gou, Qi Wang, Shufa Du, Chang Su, Jiguo Zhang, Ju-Sheng Zheng, Huijun Wang, Bing Zhang

**Affiliations:** National Institute for Nutrition and Health, Chinese Center for Disease Control and Prevention; National Institute for Nutrition and Health, Chinese Center for Disease Control and Prevention; Zhejiang Key Laboratory of Multi-Omics in Infection and Immunity, Center for Infectious Disease Research, School of Medicine, Westlake University; Chaoyang District of Beijing Centre for Disease Control and Prevention; Department of Nutrition and Carolina Population Center, University of North Carolina at Chapel Hill; National Institute for Nutrition and Health, Chinese Center for Disease Control and Prevention; National Institute for Nutrition and Health, Chinese Center for Disease Control and Prevention; Zhejiang Key Laboratory of Multi-Omics in Infection and Immunity, Center for Infectious Disease Research, School of Medicine, Westlake University; National Institute for Nutrition and Health, Chinese Center for Disease Control and Prevention; National Institute for Nutrition and Health, Chinese Center for Disease Control and Prevention

**Keywords:** Multi-trajectories, body mass index, waist circumference, dyslipidemia, gut microbiota, serum metabolites

## Abstract

**Background:**

Evidence is insufficient to establish a longitudinal association between combined trajectories of body mass index (BMI) and waist circumference (WC) and dyslipidemia. Our study aimed to explore the association between multi-trajectories of BMI and WC and incident dyslipidemia and identify microbiota and metabolite signatures of these trajectories.

**Methods:**

Stratified by sex, we used a group-based trajectory modeling approach to identify distinct multi-trajectories of BMI and WC among 10,678 participants from the China Health and Nutrition Survey over a 24-year period. For each sex, we examined the associations between these multi-trajectories (1991–2015) and the onset dyslipidemia (2018) using multivariable logistic regression adjusting for sociodemographic and lifestyles factors. We characterized the gut microbial composition and performed LASSO and logistic regression to identify gut microbial signatures associated with these multi-trajectories in males and females, respectively.

**Results:**

We identified four multi-trajectories of BMI and WC among both males and females: Normal (Group 1), BMI&WC normal increasing (Group 2), BMI&WC overweight increasing (Group 3), and BMI&WC obesity increasing (Group 4). Among males, Group 2 (OR: 2.10, 95% CI: 1.28–3.46), Group 3 (OR: 2.69, 95% CI: 1.56–4.63) and Group 4 (OR: 3.56, 95% CI: 1.85–6.83) had higher odds of developing dyslipidemia. However, among females, only those in Group 2 (OR: 1.54, 95% CI: 1.03–2.30) were more likely to develop dyslipidemia. In males, compared with Group 1, we observed lower alpha-diversity within Groups 2,3, and 4, and significant beta-diversity differences within Groups 3 and 4 (p 0.001). We also identified 3, 8, and 4 characteristic bacterial genera in male Groups 2, 3 and 4, and 2 genera in female Group 2. A total of 23, 25 and 10 differential metabolites were significantly associated with the above genera, except for Group 2 in males.

**Conclusions:**

The ascending combined trajectories of BMI and WC are associated with a higher risk of dyslipidemia, even with normal baseline levels, especially in males. Shared and unique gut microbial and metabolic signatures among these high-risk trajectories could enhance our understanding of the mechanisms connecting obesity to dyslipidemia.

## Background

Dyslipidemia is a major risk factor for cardiovascular disease and a leading cause of death globally, accounting for 46.7% and 44.3% of total deaths in rural and urban areas in China in 2019^[1]^. Given the increasing global burden and prevalence of dyslipidemia, it is imperative to uncover novel risk factors to prevent the occurrence and development of dyslipidemia.

Obesity is a major driver of dyslipidemia. Numerous studies have explored the associations between individual obesity indicators and dyslipidemia. Individuals with higher body mass index (BMI) or waist circumference (WC) are more likely to develop dyslipidemia^[2–5]^. BMI, measured by combining weight and height, is widely used to assess obesity, but it cannot capture the distribution of abdominal adipose tissue as WC does. Furthermore, BMI and WC can change throughout the lifespan, and several longitudinal studies have assessed the possible effects of long-term changes in anthropometric indices on the risk of dyslipidemia^[6–8]^. To our knowledge, there are no longitudinal studies reporting the relationship between multi-trajectories of BMI and WC from early adulthood and dyslipidemia among the Chinese population.

Emerging evidence suggests a close connection between the gut microbiome and both human obesity^[9–11]^ and dyslipidemia^[12, 13]^, suggesting that the gut microbiome may play an important role in the obesitydyslipidemia relationship. However, most studies on the association between BMI/WC and gut microbiome still remain at the cross-sectional level. The longitudinal effects of the BMI/WC on the gut microbiome and related metabolites are unclear. Specific gut microbial signatures that demonstrate patterns of BMI/WC changes over time could help explain mechanistic links between obesity and dyslipidemia.

Therefore, in the present prospective cohort study followed from 1991 to 2018, we established multi-trajectories based on 24 years of BMI and WC measurement data and revealed the gut bacterial genera and serum metabolites associated with those multi-trajectories.

## Methods

### Study population

This study is based on the China Health and Nutrition Survey (CHNS), an ongoing population-based longitudinal study. The CHNS collects demographic information, lifestyle details, physical activity levels, dietary habits, anthropometric measurements, and biological samples ^[14]^. Across 11 survey rounds, approximately15,000 participants were recruited in each round, representing 16 provinces and megacities across China. The most recent data available is from the 2018 survey.

**Figure 1:**
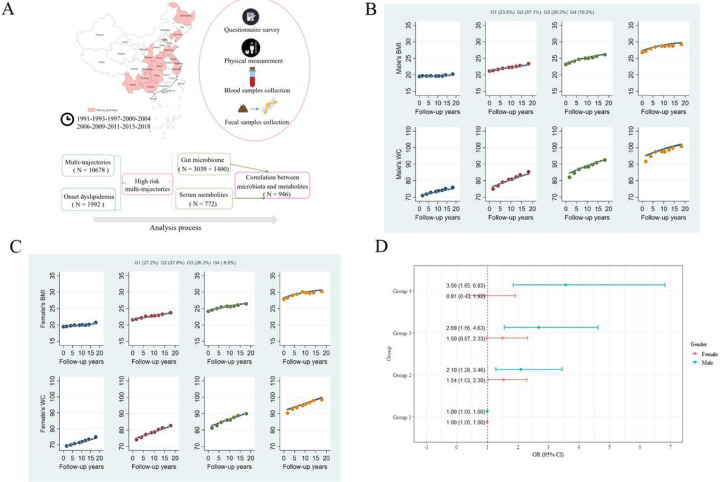
Multi-trajectories of BMI and WC and their associations with dyslipidemia. **(A)** Summary of the study population.**(B&C)** Multi-trajectories of BMI and WC in CHNS cohort (1991–2015) among males **(B)** and females **(C)**. The solid lines represent average estimated BMI and WC over time. The dots represent the actual data, where we weighted each individual’s responses based on posterior probabilities of group membership. **(D)** Associations between multi-trajectories and dyslipidemia based on binomial logistic regression model. Both models were adjusted for age, location (urban/rural), geographical area (province), education level, smoking, drinking, household income, physical activity, and dietary energy intake.

According to the analysis process, our study included five sub-datasets (Fig. 1A). (1) After excluding participants < 18 years of age, pregnant or breastfeeding, or patients with cancer, stroke or disabled patients at the time of the survey, 10,678 individuals (5,222 males and 5,456 females) with at least three weight, height and WC measurements from 1991 to 2015 were included in the multi-trajectories analysis. (2) Among these participants, we further excluded those who had developed dyslipidemia in 2015 or did not continue to participate in the cohort study in 2018 (no lipid data in 2018), leaving 1,992 individuals (841 males and 1151 females) analyzed the association between the multi-trajectories and onset dyslipidemia in 2018. (3) When analyzing the relationship between multi-trajectories and gut microbiome, we included participants with stool samples collected in the trajectory population in 2015, and excluded those who had taken antibiotics within 3 months, used probiotics within the last 4 weeks, or had gastrointestinal disorders, diarrhea, and intestinal resection. 3,039 individuals (1434 males and 1605 females) were included in the discovery cohort, and 1,400 individuals (650 males and 750 females) were included in the validation cohort. (4) A total of 772 participants (334 males and 438 females) with both multi-trajectories and metabolic data in 2015 were used to analyze the differences in metabolites between multi-trajectories. (5) We selected 946 individuals (393 males and 553 females) as a subset of the validation cohort who had gut microbiome and metabolic data in 2015 to map the connection between metabolites and gut bacteria.

### Data collection

#### Questionnaire survey

Sociodemographic characteristics, including location (urban or rural), geographical area (province), age, sex, education and household income, lifestyle factors (smoking status and alcohol consumption), dietary intake, physical activity, physiological and disease status (pregnancy, lactation, disability, stroke, cancer, gastrointestinal disorders, diarrhea, and intestinal resection), and medication data (the use of antibiotics and probiotics) were collected by face-to-face questionnaire interviews. Dietary energy intake was calculated by combining food intake data with the China food composition table, and the amount of physical activity was calculated by multiplying the amount of exercise time by activity intensity of various intensities.

#### Physical measurement

Anthropometric data, including height, weight and waist circumference, were measured on-site by trained staff. Adhering to consistent measurement standards and utilizing specialized instruments, in each survey, our trained physicians and nurses measured height and weight without shoes to the nearest 0.1 cm and 0.1 kg. We then calculated BMI as weight in kg divided by height in meters (m) squared (kg/m^2^). We measured waist circumference using an inelastic soft ruler with a division value of 0.1 cm.

#### Biological sample collection

Fasting blood samples were collected, and stored in dry ice, and sent to the laboratory for storage at −80°C within 3 hours. The plasma was centrifuged within 48 hours and stored at −80°for later use. Fecal samples were collected following standard procedures^[15]^ and temporarily stored in a −20°C freezer within 20 minutes and then stored in a laboratory − 80°C freezer.

#### Assessment of dyslipidemia

Total cholesterol (TC), triglyceride (TG), high-density lipoprotein cholesterol (HDL-C), and low-density lipoprotein cholesterol (LDL-C) were measured using an automatic biochemical analyzer. The definition of dyslipidemia can be found in the Guidelines for the Prevention and Treatment of Dyslipidemia in Chinese Adults (2016 revised edition), including the flowing thresholds: TC ≥ 6.2 mmol/L, TG ≥ 2.3 mmol/L, LDL-C ≥ 4.1 mmol/L, or non-HDL-C ≥ 4.9 mmol/L.

#### Bioinformatics analysis of gut microbiome

Methods of DNA extraction, amplification, and sequencing have been described previously^[16]^. Taxonomic and functional profiles were generated using the Quantitative Insights Into Microbial Ecology 2 platform (QIIME2)^[17]^. Pair-end reads were assembled using the QIIME tools import command. Low-quality regions of the sequences, marker gene Illumina sequences, and chimeric sequences (“consensus”) were filtered using the DADA2 pipeline^[18]^. Reads were then summarized to amplicon sequence variants (ASV) in a feature table and annotated based on the naive Bayes classifier using the classify-sklearn package against the Silva-132–99 reference sequences^[19]^.

#### Serum metabolome analysis

For metabolic analysis, 50 μL of the sample and 300 μL of the extraction solution (ACN: Methanol = 1:4, V/V) containing internal standards were added to a 2 mL microcentrifuge tube. The sample was vortexed for 3 minutes and then centrifuged at 12,000 rpm for 10 minutes at 4°C. Subsequently, 200 μL of the supernatant was collected and placed at −20°C for 30 minutes, followed by another centrifugation at 12,000 rpm for 3 minutes at 4°C. An aliquot of 180 μL of the supernatant was transferred for LC-ESI-MS/MS analysis. The sample extracts were analyzed using an LC-ESI-MS/MS system (UPLC, ExionLC AD, https://sciex.com.cn/; MS, QTRAP^®^ System, https://sciex.com/) following standard protocols. The triple quadrupole-linear ion trap mass spectrometer (QTRAP) was used to perform LIT and triple quadrupole (QQQ) scans, operated and controlled by Analyst 1.6.3 software (Sciex) with standard parameters. The source temperature was 500°C; the ion spray voltage (IS) was 5500 V (positive) and 4500 V (negative); the ion source gas I (GSI), gas II (GSII), and curtain gas (CUR) were set at 55, 60, and 25.0 psi, respectively; the collision gas (CAD) was set to high. Instrument tuning and mass calibration were performed with 10 and 100 μmol/L polypropylene glycol solutions in QQQ and LIT modes, respectively. A specific set of MRM transitions was monitored for each period according to the metabolites eluted within this period.

### Statistical analysis

#### Multi-trajectories of BMI and WC

Group-based multi-trajectory modeling (GBTM)^[20]^ was used to determine the multi-trajectories of BMI and WC. As the WC assessment criteria are different for different sex, we performed multi-trajectory modeling with a STATA plug-in using continuous norming (cNORM) distribution for different sex^[21]^. We tested linear, quadratic, and cubic specifications for trajectory shape for participants in two, three, four, five, and six trajectory groups until we established the best-fitting model. We used statistically rigorous criteria to determine the best fit. (1) With the lowest Bayesian information criterion (BIC), we used the difference size (percentage change) of the BIC to choose between a more complex (with one additional specified trajectory group) and a simpler model; (2) we included at least 2% of the sample population in each trajectory class; and (3) we ascertained the average posterior probability value of membership within each group, where values greater than 0.7 indicate adequate internal reliability^[22]^.

#### Association between multi-trajectories and dyslipidemia

We performed logistic regression models to explore the relationship between the sex-specific multi-trajectories (1991–2015) and onset dyslipidemia (2018). We built models for males and females, and adjusted for baseline age, location (urban/rural) and geographical area (province), smoking, alcohol use, education, household income, dietary energy intake and physical activity. Then, we used the“forestplot” and “ggplot2” functions in R to plot the forest plot and display the results of the model. We considered a two-sided p value < 0.05 to be statistically significant.

#### Gut microbiome analysis

We performed all gut microbiome analyses separately for different sex. Four alpha-diversity indices were calculated at a sampling depth of 6000: Shannon’s diversity index, Observed features, Pielou’s species evenness measure, and Faith’s phylogenetic diversity. In order to display the results of the four indicators on the same axis, we used the scale function in R to standardize them, then used the Wilcoxon test to compare these indicators between the dyslipidemia risk trajectory group and the normal group, and finally displayed the results in the form of box graphs. At the genus level, using the “vegdist” function from the R package “vegan”, we calculated Bray-Curtis distances between samples using genera abundance and visualized using PCoA. We then performed a permutational multivariate analysis of variance (PERMANOVA) based on the Bray-Curtis distance to determine whether there were differences between groups.

Before identifying the characteristic genera, we first preprocessed the raw genera abundance data. Genus with a presence lower than 10% were excluded, and a centered log-ratio (CLR) transformation was applied. Two cohorts were used, including the discovery cohort (3039 samples, 1434 males and 1605 females) and the validation cohort (1400 samples, 650 males and 750 females). The selection of characteristic genera for those multi-trajectories with a higher risk of dyslipidemia was based on a discovery cohort derived from Least Absolute Selection and Shrinkage Operator (LASSO) regression. LASSO regression can simplify the model by adding a penalty function and continuously compressing the coefficient to avoid collinearity and overfitting. Important variables can be efficiently screened with a smaller sample size^[23]^. Validations of these characteristic genera were based on both the discovery cohort and the validation cohort by logistic regression. To correct for multiple testing issue, p value was adjusted by the false discovery rate (FDR) method.

#### Serum metabolites analysis

Sex-specific metabolomics analyses were also performed. Wilcoxon test was used to identify differential metabolites between different multi-trajectory groups. The ratio of the median of the comparison group to the control group was used as the Fold Change (FC) value. The selection of differential metabolites was based on the following criteria: p value < 0.05 and |log2FC| > 0.5. Raw target metabolite data were log-transformed and standardized before analysis. Relationships between these differential metabolites and the characteristic microbiota of multi-trajectories were analyzed using Spearman correlation. All differential metabolites and correlation results were visualized as volcano maps and heat maps by the ggplot2 package in the R language, respectively. When the FDR adjusted p value was < 0.05, a statistical difference was considered.

## Results

### Sample characteristics

Table 1 presents baseline characteristics of the 10,678 enrolled participants (51.1% females). The mean (±SD) age of males and females was 39.6±14.1 years and 41.0±13.4 years, respectively. The mean follow-up time was 15.9±6.1 years for males and 15.8±6.3 years for females.

### Multi-trajectories of BMI and WC

Four multi-trajectories of BMI and WC among males and females were identified by GBTM (Figs. 1B and C). We named trajectories based on baseline levels (compared to adult weight criteria WS/T 428–2013) and trends. At baseline and during follow-up, 23.5% of males and 27.2% of females had BMI and WC within the normal range (BMI 18.5–23.9 kg/m^2^, WC <85 cm in males and <80 cm in females), were grouped into the normal trajectory (Group 1). 37.1% of males and 37.8% of females had normal BMI and WC at baseline and an upward trend during follow-up, were grouped into BMI&WC normal increasing trajectory (Group 2). 29.2% of males and 26.2% of females had a BMI in the overweight range (24–27.9 kg/m^2^) and WC in the precentral obesity range (males 85–89.9 cm, females 80–84.9 cm) at baseline and an upward trend during follow-up, were grouped into BMI&WC overweight increasing trajectory (Group 3). 10.2% of males and 8.8% of females had a BMI that was obese (≥ 28 kg/m^2^) and a WC that was central obese (male ≥ 90 cm, female ≥ 85 cm) at baseline and an upward trend during follow-up, were grouped into obesity increasing trajectory (Group 4).

### Multi-trajectories and dyslipidemia

Figure 1D shows the results of logistic regression analyses exploring associations between multi-trajectories and risk of dyslipidemia. Among male participants, compared with Group 1, Group 2 (OR:2.10, 95% CI:1.28–3.46), Group 3 (OR:2.69, 95% CI:1.56–4.63) and Group 4 (OR:3.56, 95% CI:1.85–6.83) were significantly associated with a higher risk of dyslipidemia after adjusting for potential confounders. However, among females, only Group 2 (OR:1.54, 95% CI:1.03–2.30) was associated with a higher risk of dyslipidemia.

### High-risk multi-trajectories and gut microbiota

**Figure 2:**
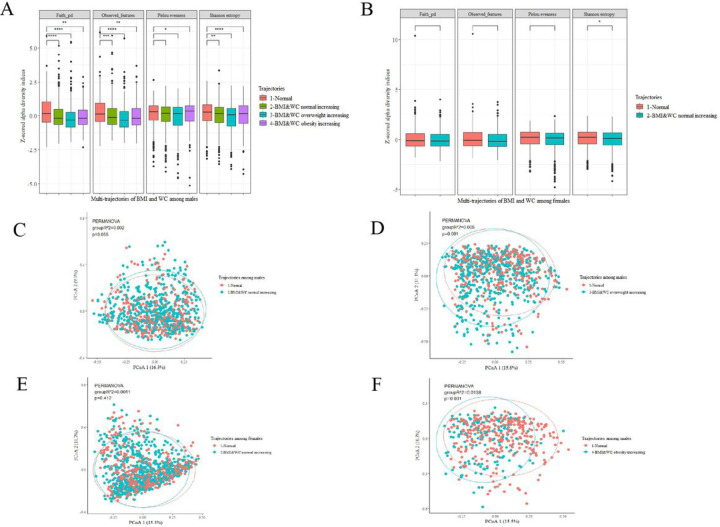
Comparison of the diversity of gut microbiota in multi-trajectories of different sex. **(A&B)** alpha -diversity analysis between males **(A)** and females **(B)**. Four alpha-diversity indices were scaled: Shannon’s diversity index, observed-features, Pielou’s measure of species evenness and faith’s phylogenetic diversity. Comparison between each risk trajectory group and the normal group using Kruskal Wallis test. **(C-F)** β-diversity analysis between males **(C, D, F)** and females **(E)**. Pairwise comparisons were determined by PERMANOVA analyses based on Bray-Curtis distance. R^2^ and p value were determined from 999 permutations.

First, we evaluated overall indicators of gut microbiota composition. Among males, we found that the values of these four alpha-diversity indexes in Group 3 were lower than those in Group 1 (Fig. 2A). Among females, only the shannon’s index value was lower in Group 2 than in Group 1 (Fig. 2B). We also identified links between multi-trajectories and overall microbial structure (beta diversity). Among male participants, permutation multivariate ANOVA based on Bray-Curtis distance showed significant differences between Groups 3, 4 and 1, explaining 0.5% and 1.4% of the dissimilarities in the gut microbiota structure (P=0.001) (Figs. 2D & 2F).

**Figure 3:**
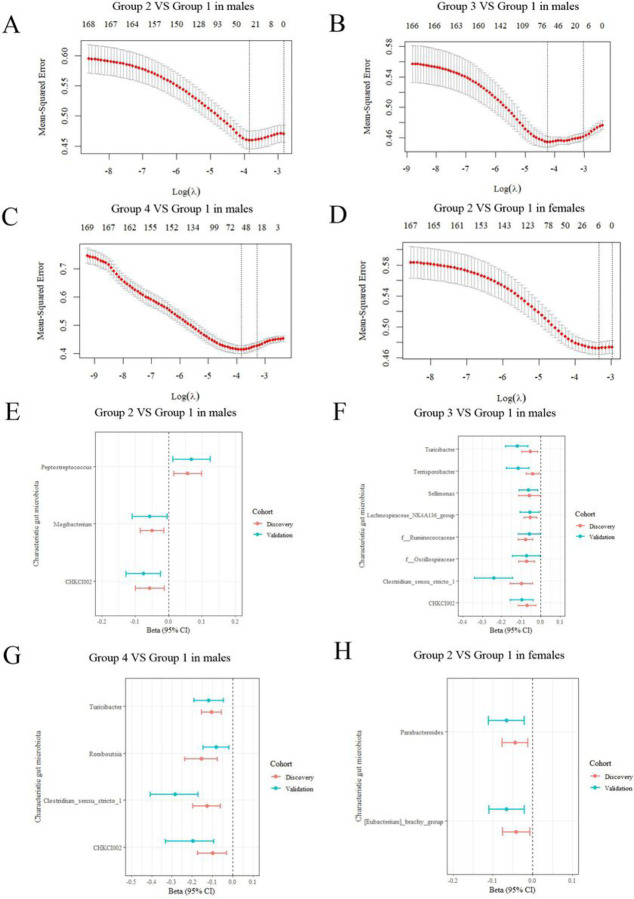
Characteristic genera for those multi-trajectories with higher risk of dyslipidemia. **(A-D)** Cross-validation curves of 169 bacteria genera for screening of characteristic genera by LASSO regression (discovery cohort). Characteristic genera selection for BMI&WC normal increasing trajectory (group 2) in male **(A)**, BMI&WC overweight increasing trajectory (group 3) in male **(B)**, BMI&WC obesity increasing trajectory (group 4) in male **(C)** and BMI&WC normal increasing trajectory (group 2) in female **(D)**. **(E-H)** Characteristic genera with statistical significance after validation in both discovery cohort and validation cohort by logistic regression. Association of selected characteristic genera with BMI&WC normal increasing trajectory (group 2) in male **(E)**, BMI&WC overweight increasing trajectory (group 3) in male **(F)**, BMI&WC obesity increasing trajectory (group 4) in male **(G)**, and BMI&WC normal increasing trajectory (group 2) in female **(H)**.

We then explored those multi-trajectories characteristic genera with a higher risk of dyslipidemia. Among males, 30, 66 and 54 microbiol genera were obtained for groups 2, 3 and 4 by LASSO regression using minimum cross-validation error as parameter in the validation dataset (Figs. 3A, 3B, and 3C). Among females, the model retained only 6 bacterial genera as characteristic genera for Group 2 (Fig. 3D). These selected bacterial genera were further validated by logistic regression. After dual verification, 3, 8, 4 and 2 characteristic bacteria genera were retained for different sex (Fig. 3E–G). Among males, both Group 3 (r = −0.10 and −0.24 in discovery and validation cohort) and Group 4 (r=−0.13 and −0.28 in discovery and validation cohort) were negatively associated with genus *Clostridium_sensu_stricto_1*, validation cohort with a higher absolute coefficient value. The same is true for Genus Turicibacter. Meanwhile, the genus CHKCI002 should also be mentioned, as all Groups 2, 3, and 4 were negatively correlated with it, and correlations were growing (Additional file 1: Table S1 −3). Among females, Group 2 was negatively associated with genus *Parabacteroides* and *[Eubacterium]_brachy_group* (Additional file 1: Table S4). The correlation was considered statistically significant when the FDR p value was less than 0.05. Detailed results are shown in Additional file 1: table S1–4.

### Key serum metabolites associated with high-risk multi-trajectories and related gut microbiota

**Figure 4:**
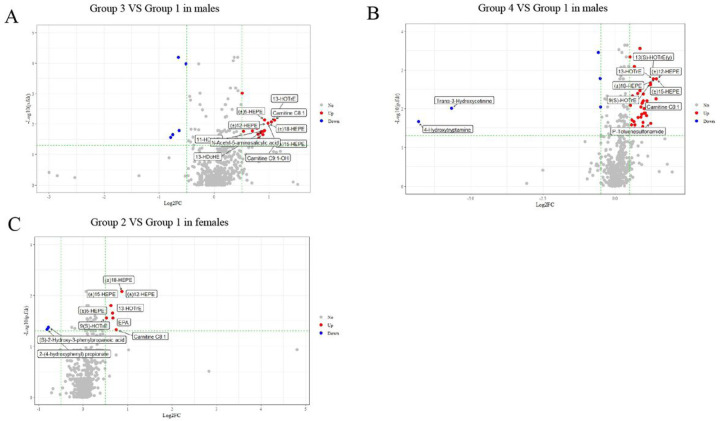
Volcanic map of differential metabolites (P<0.05, |log2(FC)>0.5 considered to be differential). Differential metabolites were found between the normal group (group 1) and BMI&WC overweight increasing trajectory (group 3) in male **(A)**, BMI&WC obesity increasing trajectory (group 4) in male **(B)** and BMI&WC normal increasing trajectory (group 2) in female **(C)**. Blue was the down-regulated differential metabolite, red was the up-regulated differential metabolite, and metabolites with no difference were marked as grey. The P value was further adjusted for multiple testing of pairwise comparison using the Benjamini-Hochberg method. Figure A and Figure B showed only the top 10 metabolites with significant differences respectively, and the top 10 metabolites were sorted according to the absolute value of log2FC.

We first identified some differential metabolites of multi-trajectories with higher risk compared with the normal group. In males, no difference was found in Group 2 metabolites (Additional file 1: Table S5). Thirty-six differential metabolites were found in Group 3, of which 30 were upregulated metabolites. For Group 4, 52 out of 57 differential metabolites were upregulated (Fig. 4A&B, Additional file 1: Table S6–7). In females, 10 differential metabolites were identified in Group 2 (Fig. 4C, Additional file 1: Table S8).

**Figure 5:**
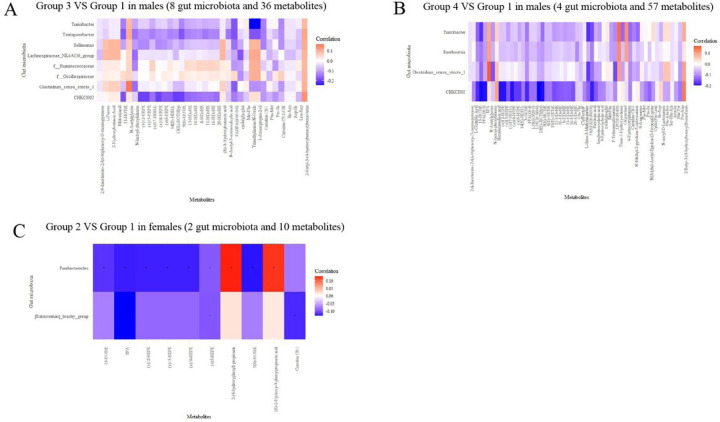
Spearman’s rank correlation between validated characteristic genera and differential metabolites. **(A)** Association of validated 8 characteristic genera for BMI&WC overweight increasing trajectory (group 3) with 36 differential metabolites (between group 3 and group 1) in male. **(B)** Association of validated 4 characteristic genera for BMI&WC obesity increasing trajectory (group 4) with 57 differential metabolites (between group 4 and group 1) in male. **(C)** Association of validated 2 characteristic genera for BMI&WC normal increasing trajectory (group 2) with 10 differential metabolites (between group 2 and group 1) in female.

We then examined the associations between characteristic microbiota (n = 8 in Group 3, = 4 in Group 4 in males, and = 2 in Group 2 in females) and differential metabolites (n = 36 in Group 3, = 57 in Group 4 in males, and = 10 in Group 2 in females) (Fig. 5). The results showed that, in males, 23 metabolites were significantly associated with the characteristic genera of Group 3. Among them, FAHFA (8:0/10:0) was negatively associated with the genera *Clostridium_sensu_stricto_1* (r=−0.17) and *Turicibacter* (r −0.18). N-lactoyl-phenylalanine was negatively correlated not only with the genera *Clostridium_sensu_stricto_1*(r −0.15), but also with two other characteristic bacteria (*Lachnospiraceae_NK4A136_group and Terrisporobacter*). Negative associations were also found among Trimethylamine-N-Oxide, 1-Aminopropan-2-ol and genera *Turicibacter, Terrisporobacter* (Fig. 5A, Additional file 1: Table S9). Twenty-five metabolites were significantly associated with characteristic genera of male Group 4. Among them, the result of FAHFA (8:0/10:0) were similar to those of Group 3. In addition, negative associations were also found among FFA (18:3), pinolenic acid and genera Turicibacter, CHKCI002 (Fig. 5B, Additional file 1: Table S10). In females, all 10 differential metabolites were significantly associated with characteristic genera in Group 2. Among them, negative associations were found among EPA, (±)5-HEPE and genera *Parabacteroides, [Eubacterium]_brachy_group* (Fig. 5C, Additional file 1: Table S11).

## Discussion

The main results of our cohort study, based on anthropometric measurements, blood and fecal samples and 27 years of data, are as follows. First, four multi-trajectories of BMI and WC among males and females were identified, with the same increasing trend from 1991 to 2015. Most of the population was in the BMI&WC normal growth group (Group 2). In addition, male participants with increasing trends in BMI and WC had a higher risk of dyslipidemia in 2018, with OR value increasing as baseline BMI and WC shifted from normal to obese. However, in female participants, we only found an increased risk of dyslipidemia in Group 2. And second, we found several important characteristic genera negatively associated with groups that remained overweight/obesity or developed overweight/obesity, including *Clostridium_sensu_stricto_1, Turicibacter*, and *CHKCI002* among males and *Parabacteroides, [Eubacterium]_brachy_group* among females. These important characteristic genera are also related to some differential metabolites, such as FAHFA(8:0/10:0), N-lactoyl-phenylalanine, Trimethylamine-N-Oxide, 1-Aminopropan-2-ol, FFA (18:3), Pinolenic acid, EPA and (±)5-HEPE; most of them belong to free fatty acids (FFAs) and oxidized lipids.

Using 24-years of BMI and WC measurements and the GBTM method, we successfully established four distinct multi-trajectories of BMI and WC in the study sample. This grouping can not only reflect the status of obesity and central obesity, but also show the baseline obesity level and long-term change trends simultaneously. To the best of our knowledge, only one study^[24]^ reported multi-trajectory of BMI and WC, and its results were similar to our study. In addition, the BMI trajectories^[25, 26]^ and WC trajectories^[27]^ derived from large samples of adults only also showed four increasing trend groups. This reminds us that the BMI and WC of Chinese adults seem to be on the rise simultaneously and need to be controlled urgently.

In our study, male groups 2, 3, and 4 were associated with a higher risk of dyslipidemia. This is different with females. A cross-sectional study among Chinese primary school children also found sex differences in anthropometric indicators predicting dyslipidemia, which may be more true for boys than for girls^[28]^. Another study of Chinese adults showed that obese males also had a higher risk of dyslipidemia than females^[29]^. This sex disparity may be due to differences in lifestyle between males and females, which are risk factors of dyslipidemia^[30]^. A possible explanation may also be related to sexual dimorphism in fat distribution and hormone levels. A study by Nedungadi and Clegg^[31]^ showed that females have more subcutaneous fat and males have more visceral fat, suggesting a greater risk of dyslipidemia. Additionally, studies have showed that females have higher leptin and adiponectin levels (considered cardiovascular-protective factors^[32, 33]^) than males^[34, 35]^. Therefore, the association between BMI and WC and the risk of dyslipidemia is weaker in females than in males. To date, we are not aware of any longitudinal study evaluating the relationship between multi-trajectories of BMI and WC and dyslipidemia. Limited evidence from cohort studies suggests that the odds of developing dyslipidemia are associated with increased BMI^[4]^ and WC^[6]^, respectively. More effective attention and interventions should be taken to manage BMI and WC to reduce the prevalence of dyslipidemia, especially in males.

Little is known about the contribution of *Clostridium sensu stricto 1* to human gut health. Two mouse experiments in 2021 and 2022 suggest it may be a novel biomarker of obesity/obesity resistance ^[36, 37]^. Our longitudinal study in adult males also confirmed this new finding, suggesting that *Clostridium sensu stricto 1* might help prevent obesity. Clostridium are known producers of butyrate^[38]^, which contributes to the integrity of the intestinal barrier, attenuates chronic inflammation by promoting regulatory T cells, and prevent pathogen proliferation ^[39]^. Therefore, loss of butyrate-producing bacteria, such as *Clostridium*, induces chronic low-grade inflammation. However, there are also conflicting results for *Clostridium sensu stricto 1* existed. Evidence from the animal feeding experiment^[40]^ and intervention trails in obese patients ^[41]^ both indicate that *Clostridium sensu stricto 1* is reduced after weight loss interventions. Similar to *Clostridium sensu stricto 1, CHKCI002* also showed a positive correlation with butyrate in ducks^[42]^. Farkas V first reported the negative correlation between *CHKCI002* genus and chicken body weight in 2022^[43]^. In this study, *CHKCI002* was also negatively associated with all risk groups in males, suggesting a beneficial effect of *CHKCI002* on obesity indicators. Many studies have linked Turicibacter to host lipid metabolism profile, but the results are inconsistent^[44–46]^. This may be a result of phenotypic diversity among Turicibacter, where hosts may experience different lipid outcomes depending on their own specific Turicibacter strains. A recent study by Lynch JB^[47]^ further identified genes capable of altering host bile acids and lipid metabolism in *Turicibacter* strains, and positioned *Turicibacter* bacteria as modulators of host lipid biology. Our data showing that *Turicibacter* a negatively associated with BMI&WC in the overweight or obesity increasing trajectory groups (two groups with higher risk of dyslipidemia) support these previous studies. This also highlights the advantage of long-term trajectory characteristic microbiota of obesity indicators in predicting dyslipidemia.

In addition to the above-mentioned beneficial bacteria found in males, we also found two dominant beneficial bacteria in females: genus *Parabacteroides* and *Eubacterium brachy group*. Members of the genus *Parabacteroides* are saccharolytic bacteria that produce major end products of fermentation, such as acetic acid and succinic acid^[48]^. According to numerous studies, the relative abundance of *Parabacteroides* is negatively associated with BMI ^[49–51]^, which is consistent with our study. There are limited studies on the association of the *Eubacterium brachy group* with obesity or blood lipids. We found only one study on adult mice showing that a high-fat diet reduced the abundance of the *Eubacterium brachy group* at 18 weeks ^[52]^. Members of the genus *Eubacterium* can undergo bile acid and cholesterol transformations in the gut, thereby contributing to its homeostasis^[53]^. Gut microbiologists agree that specific butyrate-producing microbial strains belonging to the genera *Eubacterium* may ultimately be considered as beneficial to human health as Lactobacillus and Bifidobacterium strains^[54]^.

N-lactoyl-phenylalanine (Lac-Phe) was proposed as an “exercise hormone” that suppresses appetite and adiposity in diet-induced obese mice, however, Li et al. demonstrated complete inactivity of Lac-Phe when administered orally^[55]^. In this study, the amount of Lac-Phe in the overweight/obesity increasing group was higher than that in the normal group, which is inconsistent with previous animal studies. Future work will help elucidate whether Lac-Phe can help outrun obesity. As a bioactive metabolite of the gut microbiota, trimethylamine-N-oxide (TMAO) plays a critical role in the progression of many diseases, including diabetes^[56]^, obesity^[57]^, atherosclerosis and cardiovascular risks^[58]^. In these disease states, elevated circulating TMAO concentrations are commonly observed. Meta-analyses also revealed a positive dose-dependent association between circulating TMAO concentrations and obesity^[59]^. Mechanisms that may contribute to obesity include the role of FMO 3 (TMAO producing enzyme) in obesity regulation and adipose tissue formation^[57]^, as well as increased hepatic insulin resistance and consequent obesity through increased TMAO concentrations^[60]^.

γ-linolenic acid (GLA, 18:3n-6), Pinolenic acid and EPA(the first two belong to omega-6 polyunsaturated fatty acids (PUFA), and the last one belongs to omega-3 PUFA) were significantly higher in groups 3 and 4, and were negatively correlated with the beneficial bacteria *Turicibacter*, *CHKCI002, Parabacteroides, [Eubacterium]_brachy_group* in this study. A review study showed that n-6 PUFA derived eicosanoids have pro-inflammatory effects, whereas n-3 PUFA derived eicosanoids have anti-inflammatory activities^[61]^. Research by Sunhye Shin^[62]^ further found that the high n-6:n-3 ratio of linoleic acid-rich oil increased lipogenesis and reduced lipid oxidation and thermogenesis. More importantly, adequate intake of n-3 PUFA can significantly influence the effects of n-6 PUFA on lipoprotein profiles^[63]^. Therefore, the ratio of n-6 PUFA to n-3 PUFA is more important than the amount of a single n-6 PUFA or n-3 PUFA. This study found that overweight and obese groups have higher concentrations of n-3 and n-6 fatty acids and a higher risk of dyslipidemia, which may be related to the ratio of the two.

This study has several strengths. First, we used 24-year multi-trajectories of BMI and WC to predict the risk of dyslipidemia, and long-term follow-up is a unique strength of this study. In addition, for the first time, the characteristic gut microbiota and serum metabolites of multi-trajectories of BMI and WC with higher dyslipidemia risk were analyzed. This study also has limitations. First, the characteristic bacteria or differential metabolites in the multi-trajectories were derived from observational data and the causality cannot be established at this stage. However, we conducted validation across different datasets and compared differential metabolites by p-value and screened based on FC value. Second, although we adjusted for numerous covariates, we cannot completely rule out the possibility of residual confounding. Third, the associations of obesity with functional profiles of the gut microbiome is unclear due to the use of 16S rRNA data.

## Conclusions

In conclusion, our results suggest that obesity indicators influence dyslipidemia in males more than in females. We also identified some potentially gut bacteria and differential metabolites associated with long-term BMI and WC trajectories, some of which were closely related to lipid levels, revealing the role of gut bacteria and related metabolites in obesity and lipid metabolism.

## Figures and Tables

**Table 1:** Baseline characteristics of the population in the multi-trajectories analysis

	Male 5222(48.9%)	Female 5456(51.1%)
Follow-up time(years)^a^	15.9±6.1	15.8±6.3
Age(years)^a^	39.6±14.1	41.0±13.4
Urban(%)	29.7	31.8
Education(%)		
Primary and below	39.7	56.3
Junior high	35.7	26.6
Senior high and above	24.6	17.1
Household income(RMB)^b^	1356.5(629.7,3220.6)	1378.1(636.7,3393.6)
Smoker(%)	64.4	4.7
Alcohol drinker (%)	63.9	11.5
Energy intake(kcal/d)^a^	2594.1±749.2	2259.4±670.3
Physical activity(METs/week)^b^	292.0(126.0,528.0)	336.2(144.1,589.9)
Body mass index(kg/m^2^)^a^	22.1±2.9	22.4±3.2
Waist circumference(cm)^a^	78.7±9.4	76.5±9.2

a Mean (SD) was reported,

b Median(Q1,Q3) was reported.

## Data Availability

The datasets generated and/or analysed during the current study are not publicly available, please contact the corresponding authors for details.
